# High Prevalence of Rare Mutations in the Beta Globin Gene in an Ethnic Group in Iran

**Published:** 2011-05-01

**Authors:** H Galehdari, B Salehi, M Pedram, M Oraki Kohshour

**Affiliations:** 1 Department of Genetics, Shahid Chamran University,Jundishapur University of Medical Sciences, Ahvaz, Iran; 2 Thalassemia and Hemoglobinopathies Research Center,Jundishapur University of Medical Sciences, Ahvaz, Iran; 3 Toxicology Research Center,Jundishapur University of Medical Sciences, Ahvaz, Iran; 4 Department of Immunology, School of Medicine,Jundishapur University of Medical Sciences, Ahvaz, Iran

**Keywords:** Beta thalassemia, Eizeh, Baq-Malek, Iran, Bakhtiari, Mutations

Dear Editor

Beta Thalassemia major is a genetic disease with an autosomal recessive pattern and is differentiated by severe microcytic hypochromic hemolytic anemia with hepatosplenomegaly, ineffective erythropoiesis and bone marrow expansion.[1] The β-globin (HBB) gene coding for β-chain expression is located on a 50 kb globin gene cluster on the short arm of chromosome 11, whose mutation mediated inactivation is usually responsive for the outcome of disease. Beta thalassemia occurs frequently in the coastline areas of the world. The Iran, with approximately 25000 beta thalassemia major patients and two millions expected carriers and an overall carrier frequency of 4-8%, counts to the Middle East countries with the highest prevalence of beta thalassemia.[2] The Khuzestan Province in the southwest Iran with approximately 4.000.000 inhabitants ranks to the regions with the higher prevalence of the beta thalassemia, the most common type of hemoglobinopathies in Iran.[3] Moreover, the cities Eizeh with nearly 120.000 and Baq-Malek with 25.000 citizens are located in the North Khuzestan and are categorized to the small cities with relatively high carriers of the beta thalassemia.

In the last decade, numerous studies have been published about distribution and frequency of mutations within the HBB gene in some regions of the Iran.[4][5] These data were used to manage and provide a country-wide network for detecting beta thalassemia carrier and major individuals, which need intensive medical care. Nowadays, there are centers with special task to perform prenatal molecular diagnosis of the beta thalassemia in Iran with the goal to radically reduce the transfusion dependent beta thalassemia patients. However, the success of such centers is depending on the expanding knowledge about the kind and the continuation of pathogenic mutations within the ethnic groups in different area of the country.[6]

We firstly attempted also to fully analyze the beta globin gene in 130 blood transfusion dependent individuals in the two closely located towns, namely Eizeh and Baq-malek, After investigation a cohort of 200 beta thalassemia major patients and more than 1000 carriers in the Khuzestan Province in a previous study,[3] where we showed more than 40 mutations within different ethnic groups in the province. Currently, we would know, whether the pure ethnic group such as Bakhtiari suggests similar frequency and distribution of mutations in the HBB gene as the province-wide study indicated.

A total of 260 beta-globin alleles resulting from 130 blood transfusion-dependent patients (beta thalassemia major) were studied. All of analyzed patients belonged to the Bakhtiari ethnicity. Full sequencing of the beta globin gene in the subjected individuals revealed 15 β-thalassemia causing mutations as have been listed in the Table 1. Fifty eight patients were homozygous and 72 patients were compound heterozygous for mutations. The seven most frequent mutations constituted 83.92% of all mutations. The mutations CD36/37(-T) and the IVS-II-1 were with 22.70% and 19.23% the most common mutations, accordinely.

**Table 1:** Frequency of detected mutations in the ethnic group from Eizeh and Baq-Malek in comparison to other provinces in Iran.

**Mutations**	**Type**	**N (allele)**	**Eizeh and Baq-Malek (in %)**	**Khuzestan (in %)**	**Mazandaran and Gilan (in %)**	**Origin**
^CD36/37 -T^	β^0^	59	22.70	20.54	0.8	Kurdish .Iranian
IVSII-1(G>A)	β^0^	50	19.23	20.01	56.1	Mediterranean, US Blacks
5/UTR +20(C>T)^a^	β^+^	30	11.53	1.00	-	Iranian
BETA -28 (A>C)	β^+^	27	10.38	2.10	0.5	Kurdish
IVSI-110(G->A)	β^+^	22	8.46	14.18	2.5	Mediterranean
CD8 (-AA)	β^0^	16	6.15	3.00	1.5	Mediterranean
BETA -88(C>A)	β^+^	15	5.77	0.82	0.3	Kurdish
IVSI(-del 25nt)	β^0^	8	3.06	2.54	1.2	Med East
IVSII-745[C>G] a	β^+^	30	11.53	1.30	2.3	Mediterranean
CD44 (-C)	β^0^	6	2.30	1.73	-	Kurdish
IVSI-1 (G>A)	β^0^	5	1.92	3.73	0.8	Mediterranean
CD22/23/24 (-AAGTTGG)	β^0^	5	1.92	0.64	3.0	Turkish
INITIATION CODON[T>C]	β^0^	5	1.92	0.36	-	East Asia .Med
IVSI-5(G->C)	β^+^	4	1.54	5.18	2.3	Asian Indian, SE Asian, Melanesian
CD39 (C>T)	β^0^	4	1.54	3.50	-	Mediterranean
CD5 (-CT)	β^0^	4	1.54	4.63	0.5	Mediterranean
TOTAL	-	260	100	-	-	-

^a^ both mutations occurred always in cis.

In addition, we found in 11.53% of cases (No=30), the mutation 5UTR+20 in association with the IVSII-745 on one allele ( in cis) either compound with the 3th mutation on the second allele (N=12) or in hemozygous manner (in 9 cases; N=18). The beta thalassemia major individuals were also subjected for screening of the HBB gene in the two neighboring cities in the north-east Khuzestan Province because of some reasons; both cities harbor the ethnic group “Bakhtiari” with less than 300.000 inhabitants, which show relatively high prevalence of beta thalassemia carriers.[5] Comparative studies were performed from different provinces in the Iran,[1][2][3][4][5] but just few investigations were particularly based on the ethnic groups (e.g. Arab, Tork, Lor, etc.).

In general, a minor part of thalassemia mutations predominant for geographic regions or countries.[6][7][8] We showed in a previous province-wide study more than 40 mutations and in the present study 14 different mutations with similar trends regarding the distribution and the frequency. In contrast, we did find a mutation at 5/UTR (+20) with more than 11%, which was reported in the province and in the country-wide study with 1% as a rarely event.[3] According to the previous reports, the mentioned mutation occur usually in cis with the mutation IVSII-745(C>G) and the most prevalence of the mentioned mutation was reported in Turkish Cypriots with 6.07% and in Sicilian with 6.16%.[9][10] We propose it therefore for founder mutation, at least in Iran, because of its rare detection rate in other regions of the country. Nevertheless, the hypothesis of founder effect has to be proven by genealogical studies in the target population. Such studies about carrier frequency and origin of mutations in the specific regions would be beneficial to better understand the rout and expansion of pathogenic mutations in the HBB gene.

## References

[1] Mehrabani D, Pasalar M, Afrasiabi AR, Mehravar Z, Reyhani I, Hamidi R, Karimi M (2009). Frequency of Thalassemia, Iron and Glucose-6Phosphate Dehydrogenase Deficiency Among Turkish Migrating Nomad Children in Southern Iran. Acta Medica Iranica.

[2] Najmabadi H, Karimi-Nejad R, Sahebjam S, Pourfarzad F, Teimourian S, Sahebjam F, Amirizadeh N, Karimi-Nejad MH (2001). The β-thalassemia mutation spectrum in the Iranian population. Hemoglobin.

[3] Galehdari H, Salehi B, Azmoun S, Keikhaei B, Zandian KM, Pedram M (2010). Comprehensive Spectrum of the β-Thalassemia Mutations in Khuzestan, Southwest Iran. Hemoglobin.

[4] Rahim F, Abromand M (2008). Spectrum of ß-Thalassemia mutations in various Ethnic Regions of Iran. Pak J Med Sci.

[5] Karimi M, Mehrabani D, Pasalar M, Afrasiabi AR, Mehravar Z, Reyhani I, Hamidi R (2010). Thalassemia, iron and g6pd deficiency in Lor migrating nomad children,Southern Iran. Iran Red Crescent Med J.

[6] Najmabadi H, Pourfathollah AA, Neishabury M, Sahebjam F, Krugluger W, Oberkanins C (2002). Rare and unexpected mutations among Iranian beta-thalassemia patients and prenatal samples discovered by reverse- hybridization and DNA sequencing. Haematologica.

[7] de Vooght KM, van Wijk R, van Solinge WW (2009). Management of gene promoter mutations in molecular diagnostics. Clin Chem.

[8] Kazazian HH Jr, Dowling CE, Waber PG, Huang S, Lo WH (1986). The spectrum of beta-thalassemia genes in China and Southeast Asia. Blood.

[9] Oner R, Altay C, Gurgey A, Aksoy M, Kilinç Y, Stoming TA, Reese AL, Kutlar A, Kutlar F, Huisman TH (1990). Beta thalassemia in Turkey. Hemoglobin.

[10] Sgourou A, Routledge S, Antoniou M, Papachatzopoulou A, Psiouri L, Athanassiadou A (2004). Thalassaemia mutations within the 5'UTR of the human beta-globin gene disrupt transcription. Br J Haematol.

